# Remote Eye Movement Desensitization and Reprocessing Treatment of Long-COVID- and Post-COVID-Related Traumatic Disorders: An Innovative Approach

**DOI:** 10.3390/brainsci14121212

**Published:** 2024-11-29

**Authors:** Samuele Russo, Francesca Fiani, Christian Napoli

**Affiliations:** 1 Department of Psychology, Sapienza University of Rome, Via Dei Marsi 78, 00185 Roma, Italy; 2 Department of Computer, Control and Management Engineering, Sapienza University of Rome, Via Ariosto 25, 00185 Roma, Italy; fiani@diag.uniroma1.it (F.F.); cnapoli@diag.uniroma1.it (C.N.); 3 Institute for Systems Analysis and Computer Science, National Research Council, Via dei Taurini 19, 00185 Roma, Italy; 4 Department of Computational Intelligence, Czestochowa University of Technology, Ul. Gen. J. H. Dąbrowskiego 69, 42-201 Czestochowa, Poland

**Keywords:** EMDR, COVID-19, long-COVID, post-COVID, PTSD, trauma, remote psychotherapy, online therapy, artificial intelligence

## Abstract

**Background/Objectives:** The COVID-19 pandemic has led to increased mental health issues, particularly among long-COVID patients, who experience persistent symptoms post-recovery, potentially leading to chronic conditions. The psychological impact of long-COVID is still largely unknown, but it may contribute to mental disorders like Post-Traumatic Stress Disorder (PTSD). Given the global rise in anxiety and depression, exploring therapies like Eye Movement Desensitization and Reprocessing (EMDR) for long-COVID traumatic disorders is crucial. This study explores the effectiveness of remote EMDR therapy for PTSD-like symptoms in long-COVID conditions (LCC), assessing their emergence, the impact of LCC on mental health, and identifying key commonalities. It also examines the potential advantages of an artificial intelligence (AI)-powered platform for EMDR treatments for both therapists and patients, evaluating the response differences between remote and in-person treatment. **Methods:** We enrolled a total of 160 participants divided into two groups of 80, with the experimental group receiving EMDR treatment for PTSD-like symptoms via a remote AI-powered platform, and the control group receiving traditional in-person therapy. We compared the ANOVA for Subjective Units of Disturbance (SUDs) scores, PTSD Checklist for DSM-5 (PCL-5) scores, and Impact of Event Scale-Revised (IES-R) scores between our two groups for three cases: pre-treatment, post-treatment, and decrement. **Results:** Statistical significance analysis showed a consistent absence of significant differences between online AI-powered platforms and traditional in-presence sessions. This effectively confirms our hypothesis and highlights that no significant differences were observed between the two groups. **Conclusions:** The AI-supported remote platform demonstrates comparable efficacy in delivering EMDR therapy, confirming its potential as an effective alternative to traditional in-person methods while providing added advantages in accessibility and adaptability (e.g., remote areas, hikikomori, natural disasters).

## 1. Introduction

Long-COVID conditions (LCCs), also known as post-COVID conditions (PCCs), is a term used to describe the long-term effects experienced by some individuals after acute COVID-19 infection. These effects can persist for weeks, months, or even years [1]. The World Health Organization (WHO) defines Long COVID as starting three months after the initial COVID-19 infection [2,3]. Symptoms of Long COVID can include fatigue, brain fog, dizziness, gut problems, heart palpitations, changes in smell or taste, thirst, chronic cough, chest pain, muscle twitching, and the worsening of symptoms after any type of physical or mental exertion [4]. Long COVID can have a significant impact on mental health. It has been linked to fatigue, sleep disturbances, depression, anxiety, cognitive impairment, and Post-Traumatic Stress Disorder (PTSD), among other conditions, often diagnosed also with the support of EEG-based approaches [5,6,7]. PTSD is a disorder in which someone experiences intense, disturbing thoughts and feelings for long periods following a traumatic event [8]. In the context of Long COVID, PTSD can occur in patients who have had near-death experiences or hospitalizations related to their COVID infections, and in those who have lost loved ones to the virus and may have survivor’s guilt [9,10].

### 1.1. Long COVID and PTSD Treatment

The treatment of Long COVID and related traumas and PTSD is multifaceted. No single treatment has been proven effective, but some options are available [11]. These can include trauma therapy, Cognitive Behavior Therapy (CBT) for establishing new behaviors like sleep hygiene, and Acceptance and Commitment Therapy (ACT) for those struggling with the uncertainties of their illness [12]. Eye Movement Desensitization and Reprocessing (EMDR) is one such treatment that has shown promise because it addresses trauma at its core, allowing the brain to reprocess distressing memories through bilateral stimulation, which reduces their emotional intensity and impact. EMDR has been specifically developed to reduce intrusive traumatic memories, which are hallmark symptoms of PTSD [13]. Unlike traditional talk therapies, it does not require patients to repeatedly verbalize their trauma, making it particularly accessible and less daunting for those who struggle to express or confront their experiences. Our study demonstrates that EMDR is just as effective when delivered remotely via AI-powered platforms as it is in person, which is groundbreaking for accessibility, especially for individuals in remote areas or with mobility limitations. This approach not only broadens access, but also ensures continuity of care in challenging circumstances, like during the COVID-19 pandemic. Moreover, EMDR works more quickly than many other therapies, and is effective beyond PTSD, addressing anxiety, depression, and other trauma-related conditions. Its adaptability, efficiency, and robust scientific validation makes it a cornerstone of modern mental health treatment.

A case study described how EMDR can be applied to a case of Long COVID, showing promising results [14]. However, more research is needed to further examine the effects of online EMDR for PTSD before its wider dissemination is warranted. Remote online psychotherapy has emerged as a promising approach for treating patients with LCC and PCC since it allows them to receive treatment from the comfort of their own homes [15]. This is particularly beneficial for Long COVID patients who may experience fatigue or other symptoms that make it difficult to travel [16]. In fact, in [17] the authors recently proposed an 8-week online rehabilitation program that helped long-COVID patients improve their quality of life, with less fatigue, pain, and depression after the treatment. On the other hand, as very few cities host centers for the treatment of Long COVID conditions, the availability of an EMDR practitioner is similarly not guaranteed. Online EMDR therapy can help overcome these geographical limitations, also making it possible to attend therapeutic sessions for people with difficulties in relocating. Finally, such an approach has been proven to improve the patient´s adherence and commitment of Long COVID patients [18].

### 1.2. EMDR Therapy

First introduced in 1995 by Francine Shapiro [19], EMDR has proven to be an extremely efficient treatment for PTSD-suffering patients through the administration of bilateral stimulation to the patients [20,21]. While the intrinsic mechanism behind the efficacy of the protocol has not yet been understood, it has been verified that bilateral stimulation, especially in the visual form, greatly helps the processing of traumatic memories by reducing their stressful impact on the patients [22]. The EMDR protocol follows eight precise phases, which while adaptable in case of mild symptoms [23,24,25] must be closely followed for an effective treatment [26]:

1. Phase 1: patient history, consisting of a detailed clinical interview to explore the mental state, history, and trauma of the patient;
2. Phase 2: preparation, in which the structure of EMDR therapy is explained and the methods which could lessen the impact of the traumatic memory on the patient are identified, e.g., safe place [27];
3. Phase 3: assessment, where the traumatic memory, also defined target event, is identified and the Subjective Units of Disturbance (SUDs) and the Validity of Cognition (VoC) are evaluated;
4. Phase 4: desensitization, which involves the patient focusing on a target memory while engaging in bilateral stimulation (for example by eye movement) to reduce the emotional intensity associated with the memory;
5. Phase 5: installation, where the positive belief related to the target event is strengthened;
6. Phase 6: body scan, which aims at eliminating any residual physical discomfort the patient might be experiencing;
7. Phase 7: closure, where the therapist ensures the patient is emotionally stable before concluding the session;
8. Phase 8: reevaluation, where the therapist reassesses the targeted memories to determine the effectiveness of the EMDR treatment.

Such a protocol requires the therapist to physically perform the bilateral stimulation on the patient. While tactile stimulation can be substituted by “butterfly hug”, a self-performed stimulation, visual stimulation must be administered by the therapist through hand movements, rendering it unsuitable for virtual therapy.

### 1.3. EMDR Remote Psychotherapy

One of the most recent fields of study in psychotherapy is the execution of pre-existing in-presence protocols in settings where the physical presence of a therapist cannot be guaranteed (e.g., pandemics, natural disasters). EMDR, a protocol that has proven to be effective in treating the symptoms of PTSD, can also be adapted for remote delivery. The COVID-19 pandemic, which has restricted in-person therapy, has accelerated the exploration of remote EMDR therapy. Fischer et al. [28] emphasize that adapting EMDR for remote delivery involves addressing concerns such as safety, establishing a therapeutic relationship, and implementing bilateral stimulation (BLS) effectively. Remote psychotherapy has become an increasingly popular and valuable tool in the field of mental health, offering greater access to support and well-being improvement compared to standard psychotherapy [29]. The term refers to the collective use of digital infrastructures in the form of video calls, messaging apps, online platforms, or virtual reality [30,31,32] to provide counseling and treatment. This allows therapists to connect with clients remotely, providing accessibility in several scenarios where traditional in-personal therapy might be unavailable. This creates a flexible environment in which the comfort of the patient is prioritized by tackling issues such as transportation, scheduling, or privacy. Fischer et al. [28] also notes that remote therapy enables therapists to be more flexible with session lengths, and can facilitate therapy for clients who might otherwise face logistical barriers.

This study aims to verify if, with in-person use of an online infrastructure supported by Machine Learning and with the psychotherapist actively involved, the results obtained on EMDR administration for LCC are comparable to those obtained in a classical in-presence setting. The goal of this work is, therefore, to verify the applicability of an artificial intelligence (AI)-driven practitioner support platform for remote EMDR therapy that has been proven yet quite effective for mindfulness and relaxation exercises online [33]. The mindfulness protocol in [33] showed very good results, proving the efficacy of a fully remote protocol in comparison to standard in-presence therapy and showing promise for applications to other protocols that involve visual bilateral stimulation. Moreover, some works are already available in the literature on the topic of online rehabilitation for LCC patients [34,35,36], further proving the feasibility of our study.

The experiments performed in this work closely follow the academically defined EMDR protocol in its eight phases. They have been reproduced in a virtual environment, with the desensitization phase (administered via visual bilateral stimulation) being supported by the previously mentioned PC interface through automatic detection of the patient’s engagement via eye tracking, and then compared with a control group of classical therapy patients. Fischer et al. [28] highlight that remote EMDR therapy can incorporate various methods of bilateral stimulation (BLS) beyond eye movements. For instance, the “butterfly hug” technique, where clients cross their arms over their chest and tap their shoulders alternately, has been successfully adapted for remote sessions to provide tactile BLS. However, these methods do not always work for all clients. Some patients may struggle with the “butterfly hug” due to discomfort with self-touch or being touched, some may experience motor impairments (e.g., hospitalization or other physical conditions), while others may feel embarrassed performing this movement with the therapist present. Additionally, there are challenges related to the precision of the “butterfly hug” method. Patients may have difficulty coordinating the movements of their hands and arms as required, which can impact the effectiveness of the technique. In these cases, using an alternative method of bilateral stimulation, such as visual BLS, offers several advantages, including improved precision and adaptability.

From a technical perspective, the integration of machine learning into remote EMDR therapy offers significant advantages. Remote psychotherapy has some limitations, mainly related to the latency in data transmission with current digital infrastructures. For this reason, employing machine learning tools (in particular eye tracking and distance detection) in our approach allows us to improve the quality of current platforms by promptly responding to patients’ inputs; therefore, dealing with the main drawback of virtual sessions. In particular, for EMDR remote therapy, machine learning can optimize the precision of bilateral stimulation and adapt the therapeutic process in real-time, enhancing the overall effectiveness of remote treatment. For example, eye-tracking technology supported by machine learning can monitor and analyze the client’s visual focus during the desensitization phase, ensuring that the bilateral stimulation is effectively administered. Moreover, machine learning algorithms can enhance the precision and responsiveness of virtual therapy platforms by enabling real-time adjustments of the bilateral stimulation based on the client’s engagement and emotional state. Additionally, machine learning can help address challenges such as latency in data transmission and variability in client responses by adapting the therapeutic approach dynamically. This can improve the overall efficacy of the therapy by providing more personalized and responsive treatment.

## 2. Methods

**Figure 1:**
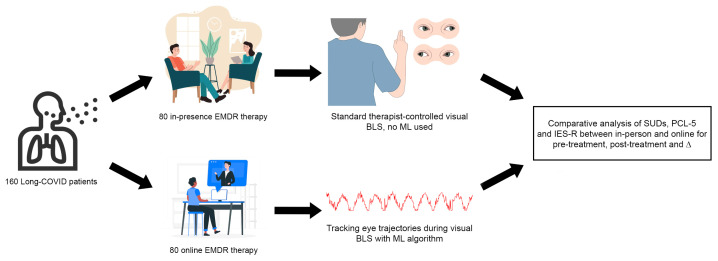
Flowchart of the study. A total of 160 subjects were randomly divided equally into two pipelines. The upper one attended standard in-presence therapy with manually performed visual BLS with no machine learning intervention. The lower one attended online therapy with ML-assisted eye tracking and virtual visual BLS. The resulting SUDs, PCL-5, and IES-R scores were compared in several conditions to verify the statistical correlation between in-presence and online therapy.

The method presented in this research involved fully online EMDR sessions, utilizing telematic infrastructures to deliver therapy remotely. The study has also used a control group composed of people who were following the standard EMDR protocol in presence. Participants were recruited through announcements posted in public spaces and hospital facilities, targeting individuals who exhibited Long COVID symptoms and related trauma. This recruitment strategy ensured the inclusion of participants meeting the criteria for PTSD or trauma-related disorders post-COVID. All participants provided informed consent before beginning the study. This section will present an overview of the design of the experiment, the sample of participants, the procedures for the online experimental group, and, finally, the metrics used for evaluation. Figure 1 briefly shows the flowchart of the study.

### 2.1. Design

All of the participants were voluntarily recruited, and to protect their anonymity, each received a randomly assigned identification code. Initially, participants were provided with an informed consent form, allowing them time to read and ask any questions. After reviewing the consent form, participants signed a copy for the records. Following the consent process, a link was sent to participants so they could anonymously complete a series of socio-anamnestic questionnaires using their assigned ID. These questionnaires were necessary to assess the inclusion criteria. Exclusion criteria included the use of psychiatric medications/substances of abuse and/or a history of psychiatric disorders or neurological conditions affecting the CNS (e.g., stroke, seizure disorder). Participants were also asked to list up to ten potentially traumatic memories, one of which would be the focus of the upcoming sessions. The form allowed for a maximum of ten memories.

Participants were then contacted to arrange the three sessions planned for the study, each lasting 45 min. In line with the study’s experimental design, participants were informed that each session needed to occur within seven days of the previous one. This guideline was intended to ensure consistency with the clinical protocols for memory processing as outlined in the standard EMDR guidelines [37]. This requirement also helped recreate a therapeutic setting similar to that of a typical psychotherapy treatment plan.

The memory to be processed was selected by the EMDR therapist based on three criteria:

1. The memory should have elicited a Subjective Units of Distress (SUDs) score between 7 and 10.
2. The memory should relate to events that could be adequately processed in three sessions, so memories with a strong relational component, such as attachment issues or those linked to personality disorders, were excluded.
3. Traumatic memories could encompass a range of emotional experiences, including loss, unresolved grief, fears related to traumatic events experienced directly or indirectly, or past situations in which the individual felt a real threat.

To address ethical concerns and ensure participant well-being, memories that could not be fully processed within the limited number of sessions were avoided. Although the study acknowledges the potential benefits of the proposed treatment for such memories, we needed to adhere to the study’s limitations to ensure scientific rigor and repeatability. Given the emphasis on participant well-being, individuals in both the standard EMDR protocol group and those participating in the online sessions were also offered the possibility of up to five total sessions if needed, with two additional sessions beyond the original three to provide extra support if necessary.

During the sessions, alternating bilateral stimulation through eye movement was employed, either in presence or during the online sessions through the developed platform, following the standard EMDR protocol. Throughout all sessions, careful attention was paid to the setup, which was specifically designed to create a comfortable and therapeutic environment. In the standard EMDR protocol setting, participants sat on a chair facing the therapist, slightly shifted to the right to facilitate eye movement. For those in the online session group, the participant’s chair was positioned in front of a computer screen, with the therapist’s chair placed behind the participant.

For both groups, the procedures followed a similar structure. In the first session, once the participant was comfortably settled, we introduced the traumatic memory that would be the focus of the treatment. The first part of the process involved gathering background information about the individual and their family history (Phase 1) to confirm that the selected memory fit the study’s criteria and could be addressed within the study’s parameters. Once the memory was chosen and verified for compatibility, the participant’s SUDs level was assessed. Participants were also asked to complete two digital questionnaires before the session began. The first, the Impact of Event Scale-Revised (IES-R), is a standardized psychometric tool with 30 items designed to measure post-traumatic symptoms. The second questionnaire, the Post-Traumatic Stress Disorder Checklist for DSM-5 (PCL-5), was used to assess symptoms of PTSD.

Following the completion of these questionnaires, participants were introduced to the EMDR process, which included an explanation of the “Stop” signal they could use to pause the alternating bilateral stimulation in the first group, or the online sessions for the second group. After this introduction, the preparation phase (Phase 2) commenced. In this phase, participants were asked to select a safe place, either real or imagined, that evoked positive and pleasant feelings. The therapist verified that the safe place chosen by the participant did not contain emotionally disturbing elements that could interfere with the reprocessing of the traumatic memory. Once verified, the safe place was reinforced in accordance with the EMDR protocol.

For participants in the in-presence EMDR group, the safe place was reinforced using slow, brief sets of eye movements. The participants in the online sessions group followed the same verbal instructions as in the EMDR protocol, but the eye movements were driven by a moving dot, with the AI-driven support system constantly verifying the correctness of the movement by tracking the eye position and informing the therapist. In both groups, participants were asked to associate a keyword with the safe place, such as “forest”, “serenity”, or “sea”, and to mentally connect the word with the safe place.

After the safe place was installed, the assessment phase (Phase 3) of the standard EMDR protocol took place. Participants were asked to recall the distressing memory and identify the most disturbing aspect of it by answering the question: “What image represents the worst part of the memory?”. They were also asked to verbalize the negative belief associated with the memory: “What words accompany the image and express a negative belief about yourself at this moment?”. To aid in this process, participants were provided with a list of negative cognitions related to three primary areas: responsibility (self-defectiveness and guilt), safety, and control over choices. Participants were also asked to identify a positive cognition: “When you think about that image, what would you like to believe about yourself right now?”. A list of suggested positive cognitions related to the same three areas was provided to help guide participants in this process. Once both the negative and positive cognitions were identified, the Validity of the Positive Cognition (VoC) was assessed. The question was: “On a scale of 1 to 7, where 1 means it feels completely false and 7 means it feels completely true, how true do you feel the positive cognition is now?”.

After identifying the emotions associated with the memory and reassessing the SUDs level, participants in the online sessions group underwent a brief training session to familiarize themselves with the system. At the end of the first session, participants were guided to recall their safe place to help them return to a state of calm before leaving.

In the second session, the reprocessing of the traumatic memory took place for both groups. Participants were reminded of the elements from Phase 3, and the use of the “Stop” signal was emphasized once again. After reassessing the SUDs level and reviewing both the negative and positive cognitions, the desensitization phase (Phase 4) began. Participants in the online sessions group received additional training to ensure they understood the task. For the “in presence” group, the instruction was to follow the therapist’s fingers with their eyes while recalling the image and the negative cognition. Both groups engaged in a series of stimulations, with the “in presence” group following eye movements and the online sessions group performing trials of variable durations to match the typical timing of EMDR stimulations.

The second session concluded for both groups, as per the in presence protocol, with recalling the safe place after the reprocessing through stimulation. Participants were informed that the processing could continue after the session and that they might experience new insights, sensations, thoughts, memories, or even dreams. If these occurred, they were encouraged to take note and discuss them in the third session. The therapist remained available to provide psychological support throughout the entire duration of the three sessions. Additionally, participants were advised to use their safe place if they felt any distress or discomfort as a result of overthinking the traumatic memory.

The third session began for both groups with a recap of the previous session, following the evaluation protocol [37], reminding participants of the content covered in the earlier session. They were also asked if they had noticed any changes after the previous session and to share their current perception of the memory that had been the focus of the work. At this point, the reevaluation of the SUDs was conducted. If the SUDs score remained above 0, the desensitization process continued; however, if the score had dropped to 0, the installation of the positive cognition followed, in line with the standard EMDR protocol. As is customary, this session also ended with a recall of the safe place and a reminder that memory processing might continue beyond the session.

Before leaving, participants were asked to complete the two questionnaires administered at the beginning of the first session (IES-R and PCL-5), this time referring to the memory they had been working on. They were also asked to report their SUDs level once again. Throughout all of the sessions, and for both groups, the therapist ensured that participants were in a psychologically stable condition before they left the treatment room. The safe place was consistently used to help participants lower their emotional arousal and return to a state of calm. Additionally, after the third session, if the therapist deemed it necessary or if requested by the participant, two more sessions could be offered for further benefit, though they would not be included in the study’s analysis.

### 2.2. Participants

There were a total of 160 participants (50% females, 50% males), aged between 25 and 49 years (m = 37.41, SD = 6.77). In the initial phase, participants were randomly allocated to one of two experimental groups: the remote AI-powered platform group (online therapy group), and the standard EM stimulation in-person group (control group). Each group comprised 80 participants. The groups exhibited minor age differences (Standard EM stimulation in-person: Mean = 37.40, SD = 6.69; Remote AI-powered platform: Mean = 37.43, SD = 6.74), while gender distribution was balanced across groups. In order to ensure that the study had sufficient power to detect a statistically significant difference between the two groups, the number of participants was determined based on a power analysis. As a result, we determined an effect size d = 0.418; therefore, the significance level was set as α = 0.05. With these parameters, considering our total sample size of 160 (80 per group) yields a power 1−β = 0.84 to correctly reject the null hypothesis if there is a true difference between the groups. This level of power is considered acceptable in many fields of research, as it balances the risks of Type I and Type II errors. The main inclusion criterion was to be affected by a set of clinical conditions compatible with PCC, and therefore compliant with the following diagnostic criteria determined by the World Health Organization (WHO) [38]:

1. A history of confirmed SARS-CoV-2 infection;
2. Unresolved symptoms after 3 months from the onset of COVID-19;
3. Symptoms lasting from more than 2 months;
4. Symptoms may also be intermittent with relapses over time.

More precisely, WHO requirements also account for unconfirmed SARS-CoV-2 infections; however, we limited our study to people who had a past infection confirmed with a positive test for COVID-19. Moreover, we limited our study to subjects between 25 and 49 years old, in order to work with a population of young adults that we could consider clinically homogeneous. The specific range was also determined, given the maximum incidence of PCC on this exact range [39]. We excluded from the study subjects with confirmed neurological or psychiatric conditions, as well as individuals with a clinical picture of depression yet before the SARS-CoV-2 infection, as well as people with suicidal thoughts.

### 2.3. Procedure

Our experiment has been constructed to study the efficacy of an innovative AI-driven online support system for EMDR approach, specifically on subjects affected by long-COVID and post-COVID-related traumas. To aid the patients during the whole process and address any doubt encountered during the sessions, a therapist and an operator were available at all times. The therapist, in particular, was performing validation during the whole duration of the BLS, verifying the correct execution of the task by the subjects and intervening in case of necessity. This effectively tackles some of the concerns regarding the application of machine learning algorithms in online psychotherapy, the two main ones being the lessened expertise of the algorithm compared to human experts and the difficulty in interactions with the system of novel users. Another concern regarding the data treatment of the subjects has also been tackled, as data has been collected according to ethical and privacy regulations. Our platform was also constructed accordingly to tackle some of the ethical concerns revolving around remote psychotherapy [40,41], with the visual recordings of patients during the therapy having only been visioned by the involved experimenters, with no employment in algorithm training. It is also important to note patients were allowed to halt the session at any noticeable discomfort, and they had no obligations to complete the treatment. For the online group, Phases 1 to 3 and Phases 7 and 8 have been carried out by means of a video call by the psychotherapist with no intervention of any machine learning algorithm. In Phases 4, 5, and 6, instead, the AI-assisted framework aided the therapist by providing automatic eye movement engagement detection and a distance detection system to guide the patient to the optimal position. The full procedure for the online group is the following:

1. Phase 1 has been carried out by means of an online interview to collect patient history and gather a comprehensive history of the patient, including past experiences, current symptoms, and any traumatic events that may be targeted during the EMDR process;
2. In Phase 2, the therapist has been working with the patient to establish a therapeutic alliance. The participants were encouraged to select a safe place that made them feel comfortable and positive, with the aim of bringing the patient back to an optimal state of arousal at the end of the processing session;
3. During Phase 3, the patient and therapist identify specific target memories or experiences to address during the treatment and evaluate the emotional distress associated with each target;
4. During Phase 4, desensitization, the patient focuses on a target memory while engaging in bilateral stimulation (for example, following a moving object with their eyes). This process helps reduce the emotional intensity associated with the memory. In in-person sessions, the therapist administers bilateral stimulation through hand movements. In contrast, during online sessions, the bilateral stimulation is facilitated by a digital interface, where the patient follows a moving visual stimulus on the screen, such as an oscillating dot. During the latter, the system has been used to analyze the subject’s adherence to the prescribed movements, offering the psychotherapist the possibility to evaluate the patient’s reactions as assessed by the eye tracking algorithm;
5. In Phase 5, positive beliefs or self-statements are strengthened and associated with the previously targeted memory during this phase, with our support system providing valuable feedback in order to evaluate the correct progression of the therapy;
6. During Phase 6, the patient addressed any residual tension or discomfort, and the eye movements are again tracked to provide feedback to the psychotherapist;
7. Phase 7 does not involve the use of artificial intelligence, with the therapist ensuring the patient is emotionally stable before concluding the session and providing coping strategies for post-treatment well-being;
8. In Phase 8, the therapist and patient reassess the targeted memories to determine the effectiveness of the EMDR treatment, with possible adjustments to address any remaining distress, without the use of AI algorithms.

Before the beginning of the therapy, the patients were debriefed on the whole therapy plan in order to familiarize them with the process and, for the online group, with the platform they would have to use. For what concerns the desensitization, the timing for the online group was identical to that of a standard in-presence EMDR session, lasting around 30 to 45 min. During these sessions, visual BLS was performed in sets of 20–30 s each, followed by an evaluation of the sensations felt by the patient. In particular, with our system, the velocity of the movement is controllable, as well as the color and shape of the dot, the number of oscillations, the direction of the oscillation, and the background color. This provides a degree of customization to the patient, which made it possible to increase their comfort during the therapy. During the visual BLS, the system tracks the distractions of the patients and provides a textual feedback to the therapist in case of distraction to prompt the patient to refocus. Moreover, the algorithm generates a graphical trajectory of the horizontal eye oscillation during each session, which can be analyzed to verify the adherence of the subjects to the prescribed movement. Subjects were asked to position themselves comfortably and move as little as possible during desensitization to reduce eye-tracking errors. Optimal positioning, which was guided by a built-in distance detection algorithm, was centered relative to the camera at a moderate distance, allowing screen-wide pupil motion without head movement. Subjects rested their backs against a chair to minimize head oscillations. The environment was optimized with lighting to aid the algorithm in eye and iris recognition, allowing subjects to keep their glasses on. No complex setup is required for this experiment, as the framework works on any standard PC.

### 2.4. Measures

The *Subjective Units of Distress Scale (SUDs)* [42,43], also known as the Subjective Units of Disturbance Scale, is a self-assessment tool that measures the subjective intensity of disturbance or distress currently experienced by an individual [43]. The scale ranges from 0 to 10, with 0 indicating peace and serenity, and 10 indicating unbearable distress. The SUDs rating is subjective, meaning it is based on the individual’s personal assessment of their distress. In the context of Eye Movement Desensitization and Reprocessing (EMDR) therapy, the SUDs are used to evaluate changes in emotion and cognition [44]. EMDR is an evidence-based treatment for Post-Traumatic Stress Disorder (PTSD) and other trauma-related disorders. During EMDR therapy sessions, the SUDs are used to gauge the intensity of the patient’s distress as they focus on the traumatic memory while simultaneously experiencing bilateral stimulation. This process is associated with a reduction in the vividness and emotion associated with the traumatic memories. The use of SUDs in EMDR therapy allows both the patient and the therapist to track improvements or setbacks in treatment. It is important to note that the precise accuracy of measurement is not as important as the general indication of the patient’s subjective experience of distress. This helps the therapist understand the severity of the patient’s emotions and adjust the treatment accordingly.

The *Impact of Event Scale-Revised (IES-R)* is a self-report measure that assesses subjective distress caused by traumatic events. It is a revised version of the older version, the 15-item IES [45]. The IES-R contains 22 items related to the symptoms of Post-Traumatic Stress Disorder (PTSD), including additional items related to the hyperarousal symptoms of PTSD, which were not included in the original IES [46]. Items correspond directly to 14 of the 17 DSM-IV symptoms of PTSD. Respondents are asked to identify a specific stressful life event and then indicate how much they were distressed or bothered during the past seven days by each “difficulty” listed. Items are rated on a 5-point scale ranging from 0 (“not at all”) to 4 (“extremely”). The IES-R yields a total score (ranging from 0 to 88), and subscale scores can also be calculated for the Intrusion, Avoidance, and Hyperarousal subscales. In the context of Eye Movement Desensitization and Reprocessing (EMDR) therapy, the IES-R can be used to evaluate changes in emotion and cognition. EMDR is an evidence-based treatment for PTSD. During EMDR therapy sessions, the IES-R is used to gauge the intensity of the patient’s distress as they focus on the traumatic memory while simultaneously experiencing bilateral stimulation [47]. This process is associated with a reduction in the vividness and emotion associated with the traumatic memories. The use of IES-R in EMDR therapy allows both the patient and the therapist to track improvements or setbacks in treatment.

The *PTSD Checklist for DSM-5 (PCL-5)* is a 20-item self-report measure that assesses the 20 DSM-5 symptoms of PTSD [48]. The PCL-5 can be used to track changes in a patient’s symptom severity during and after treatment, as well as to identify individuals who may have PTSD; in fact, the PCL-5 can be scored to provide a provisional PTSD diagnosis [49]. Similarly to SUDs, the use of PCL-5 in EMDR therapy also allows the patient and the therapist to track the efficacy of treatment. The PCL-5 is a self-report measure that can be completed by patients in a waiting room prior to a session or by participants as part of a research study. It takes approximately 5–10 min to complete. In the context of Eye Movement Desensitization and Reprocessing (EMDR) therapy, the PCL-5 can be used to evaluate changes in emotion and cognition. EMDR is an evidence-based treatment for PTSD. During EMDR therapy sessions, the PCL-5 is used to gauge the intensity of the patient’s distress as they focus on the traumatic memory while simultaneously experiencing bilateral stimulation. This process is associated with a reduction in the vividness and emotion associated with the traumatic memories. The use of both the Post-Traumatic Stress Disorder Checklist (PCL-5) and the Impact of Event Scale–Revised (IES-R) alongside the Subjective Units of Disturbance (SUDs) scale is grounded in their complementary roles in assessing trauma-related distress and therapeutic progress. The PCL-5 is a validated self-report measure designed to evaluate the presence and severity of PTSD symptoms in accordance with DSM-5 criteria [48]. Its multidimensional approach captures the full range of PTSD symptoms, including re-experiencing, avoidance, negative alterations in cognition and mood, and hyperarousal, making it a reliable tool for clinical and research purposes [49]. Similarly, the IES-R measures subjective distress related to a specific traumatic event, focusing on intrusion, avoidance, and hyperarousal symptoms [50]. It is widely used to gauge the psychological impact of trauma and track changes over time. Together, the PCL-5 and IES-R provide a robust framework for evaluating PTSD symptoms and the efficacy of interventions across different stages of treatment. The SUDs scale, originally introduced by [42] and later validated in EMDR-specific contexts [43], is a core element of the EMDR protocol. This self-reported measure quantifies the subjective distress associated with traumatic memories during the therapeutic process. It serves as an immediate, session-level indicator of emotional engagement and the reduction of distress, complementing the broader symptom-focused measures provided by the PCL-5 and IES-R. The inclusion of SUDs scores ensures consistency with established EMDR methodologies, allowing comparability with previous studies while also providing real-time insights into therapeutic progress.

## 3. Results

**Figure 2:**
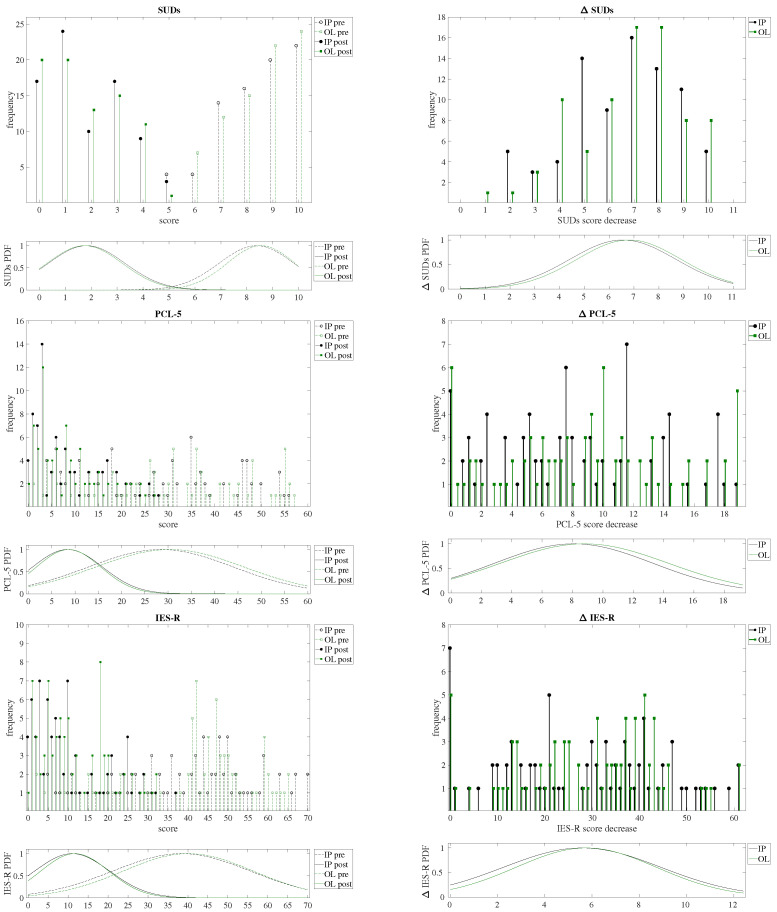
In the left column, the histograms and probability distributions (PDF) of the SUDs (**top**), PCL-5 (**middle**) and IES-R (**bottom**) tests scoring before (pre, dashed lines), and after (post, continuous line) treatment, comparing both the in-person (IP, black colored) and the online (OL, red colored) groups. On the right column the histograms and probability distributions (PDF) of the obtained decrease (Δ) after treatment. Note that, for the sake of graphical clarity, the histogram bars for the IP and OL series have been adjusted horizontally by 0.1 from the x-axis tick positions.

**Figure 3:**
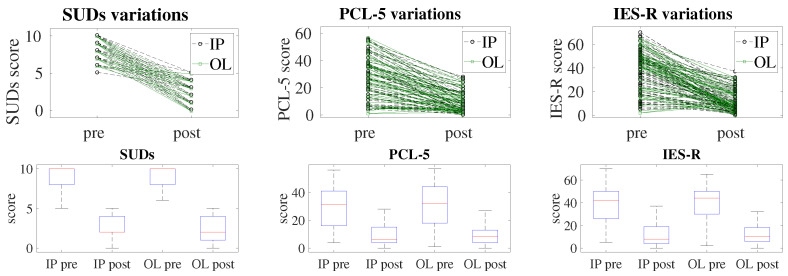
The upper panels show slope plots: the lines are used to connect the individual scores before (pre) and after (post) treatment of SUDs (**left**), PCL-5 (**middle**), and IES-R (**right**) tests, comparing both the in-person group (IP, black dashed lines) and the online group (OL, green continuous lines). A greater slope means a greater reduction in the score after treatment. Note that, for the sake of graphical clarity, the markers of the IP and OL series have been shifted away, vertically, of 0.1 with respect to the tick position on the y axis. It is possible to appreciate that there are no significant differences in slope between the groups. In the lower panels, box plots are shown: each box represents the resulting distribution for a group (in-person, IP, and online, OL), before (pre) and after (post) treatment, where the box represents the interquartile range, the line inside is the median, and the whiskers indicate the variability outside the upper and lower quartiles.

In this study, we aimed to evaluate the efficacy of EMDR therapy delivered online (OL) using an AI-aided platform devised in [33] for the treatment of post-COVID- and long-COVID-related post-traumatic disorders. We also compared our results with traditional in-person (IP) sessions. We analyzed the impact of these therapies on three primary outcome measures: Subjective Units of Distress (SUDs), Post-Traumatic Stress Disorder Checklist for DSM-5 (PCL-5), and Impact of Event Scale-Revised (IES-R). Our analysis focused on pre-treatment, post-treatment, and the change (decrease) in scores for these measures, with the results reported in Table 1, and represented graphically in Figure 2 and Figure 3.

### 3.1. Statistical Analysis

In this study, several statistical methods were used to assess the comparability and effectiveness of online (OL) and in-person (IP) EMDR therapy. These methods were chosen based on their ability to evaluate assumptions about the dataset and ensure robust analysis of the treatment effects. The rationale and purpose of each method are detailed below:

1. The *Shapiro–Wilk* and *Levene tests* to ensure that the data meets the assumptions required for parametric testing are verified.
2. The *Wald–Wolfowitz test* to confirm the randomness of the sequences, ensuring that no systematic bias affects the observations.
3. The *Analysis of Variance (ANOVA)*, for its ability to detect statistically significant differences between group means under the assumption of normality and equal variances.
4. The *Effect Size (Cohen’s d)* to quantify the magnitude of differences in treatment effects between the groups.

The Shapiro–Wilk test was conducted to verify if the residuals from the outcome measures (SUDs, PCL-5, and IES-R scores) followed a normal distribution. This test was selected because of its sensitivity in detecting deviations from normality in small to medium-sized datasets. Normality is a key assumption for parametric tests like ANOVA, ensuring the validity of the statistical inferences. The test was conducted on the residuals for the Subjective Units of Distress (SUDs), PTSD Checklist for DSM-5 (PCL-5), and Impact of Event Scale-Revised (IES-R) scores, with *p*-values of 0.072, 0.087, and 0.065, respectively. None of these values were below the significance threshold of p<0.05, indicating that the residuals did not significantly deviate from normality. To verify the homogeneity of variance, Levene’s test was used across the in-person and online groups. The results for the SUDs, PCL-5, and IES-R scores yielded *p*-values of 0.45, 0.39, and 0.42, respectively, confirming that variances were not significantly different between groups. This indicates that the homogeneity of variance assumption was met for all primary outcome measures.

Levene’s Test for Homogeneity of Variance was applied to assess the homogeneity of variances across the two groups (OL and IP). This test ensures that variances within the groups are equal, which is a critical assumption for the valid application of ANOVA. The choice of Levene’s test stems from its robustness in detecting variance differences in datasets with different sample sizes.

The Wald–Wolfowitz Test for Randomness and Independence test was used to confirm the independence of observations within the dataset. This test was chosen because it evaluates whether data points in a sequence are randomly distributed, an essential assumption to avoid biases in statistical analysis. To assess the independence of observations within the dataset, we applied the Wald–Wolfowitz test in order to evaluate the randomness of our data sequences, which can imply independence if randomness is confirmed. In our analysis, we tested the sequence of scores recorded across both the in-person and online groups to detect any patterns that might suggest dependency among observations. For the 160 observations, where the sequence contained 80 in-person and 80 online records in a randomized order, and we obtained a resulting z-score of 0.16 and a corresponding *p*-value of 0.87; therefore, the results were not statistically significant (p>0.05), indicating that the sequence of observations was random, as we wanted to demonstrate. This conclusively supports the assumption of independence among the observations in both groups. Finally, while the use of self-report scales could lead to biases, since such phenomena must equally affect both pre-treatment and post-treatment measures we can assume that biases can be neglected when comparing equally biased measures. Therefore, given the obtained results from the preliminary statistical analysis, we could then conclude that the data were appropriate for the study’s scope and suitable for ANOVA analysis.

A one-way ANOVA was conducted to compare pre-treatment, post-treatment, and improvement (decrement) scores between the two groups. This test was particularly appropriate given the design of the study, with multiple measures across two independent groups. Cohen’s d was calculated to quantify the magnitude of differences in treatment effects between the groups. Cohen’s d enhances the interpretability of the results by quantifying the size of observed effects.

### 3.2. Pre-Treatment Scores Analysis

The initial comparison of pre-treatment and post-treatment scores for both the IP and OL groups showed no significant differences as reported in Table 1. The ANOVA results for pre-treatment SUDs scores yielded an F value of 0.44 with a *p*-value of 0.51, indicating no significant difference between the IP (Mean = 8.37, SD = 1.43) and OL (Mean = 8.55, SD = 1.30) groups. Similarly, for pre-treatment PCL-5 scores, the F value was 0.59 with a *p*-value of 0.44, suggesting no significant difference between the IP (Mean = 28.43, SD = 15.55) and OL (Mean = 30.37, SD = 15.89) groups. For pre-treatment IES-R scores, the ANOVA results showed an F value of 0.51 and a *p*-value of 0.48, again indicating no significant difference between the IP (Mean = 38.41, SD = 17.24) and OL (Mean = 40.08, SD = 16.28) groups. The histograms and probability distributions of each score for both IP and OL, before (pre) and after treatment (post), are shown in Figure 2. These results confirm that the two groups were well-matched in terms of their baseline levels of distress and PTSD-related symptoms.

### 3.3. Post-Treatment Scores Analysis

Post-treatment scores also did not show significant differences between the groups, showing very similar distributions for IP and OL groups with all the collected data, thus demonstrating that both formats of EMDR therapy were equally effective. The ANOVA for post-treatment SUDs scores showed an F value of 0.1296 with a *p*-value of 0.7193, indicating no significant difference between the IP (Mean = 1.82, SD = 1.46) and OL (Mean = 1.75, SD = 1.43) groups. For post-treatment PCL-5 scores, the F value was 0.0786 with a *p*-value of 0.7797, suggesting similar outcomes for the IP (Mean = 8.38, SD = 7.49) and OL (Mean = 8.62, SD = 6.89) groups. The post-treatment IES-R scores also showed no significant difference, with an F value of 0.1924 and a *p*-value of 0.6616, indicating comparable results for the IP (Mean = 11.01, SD = 9.39) and OL (Mean = 11.68, SD = 8.52) groups. These findings suggest that both in-person and online EMDR therapies effectively reduced the symptoms of PTSD and distress to a similar extent.

### 3.4. Decrement (Δ) Analysis

The decrease in scores from pre-treatment to post-treatment, which reflects the effective improvement for a subject, also showed no significant differences between the IP and OL groups, again confirming equivalent efficacy for both IP and OL groups. For the decrease in SUDs scores, the ANOVA results yielded an F value of 1.51 with a *p*-value of 0.22, indicating no significant difference between the IP (Mean = 6.55, SD = 2.13) and OL (Mean = 6.08, SD = 2.09) groups. The decrease in PCL-5 scores had an F value of 0.89 with a *p*-value of 0.34, suggesting statistically similar levels of improvement for the IP (Mean = 20.20, SD = 13.01) and OL (Mean = 21.96, SD = 13.78) groups. The decrease in IES-R scores also showed no significant difference, with an F value of 0.09 and a *p*-value of 0.76, indicating statistically comparable reductions in symptoms for the IP (Mean = 27.90, SD = 16.65) and OL (Mean = 28.76, SD = 14.85) groups. These results reinforce the conclusion that both formats of EMDR therapy were equally effective in reducing the symptoms of PTSD and distress.

## 4. Discussion

The presented approach has proven to have many advantages, such as reducing the therapist’s fatigue in manually performing both the BLS and the eye tracking, making it possible to accurately track eye movements also from remote, and offering a tool to contrast several barriers in current psychotherapy. Some examples are:

1. More accessibility in daily life, e.g., to elderly, hikikomori, people in remote areas, etc., and in extreme scenarios, e.g., natural disasters, pandemics, war, etc.;
2. Reducing the need for therapy spaces and transportation, consequently lowering carbon emissions;
3. Allowing the patients to have sessions in a more comfortable environment, e.g., their home;
4. Extending access to therapy also for those patients who cannot leave their home due to physical, psychological, or psychopathological limitations.

Early intervention is especially important, since it has been proven that EMDR is also efficient in treating Recent Traumatic Events (RTE), with protocols such as the Recent Traumatic Episode protocol (R-TEP) being developed in the scope [51,52,53,54,55]. Thanks to the compatibility between our method and available devices, early intervention is not only possible, but also encouraged. Compared to previous works on telehealth solutions for PTSD treatment [56,57,58], our algorithm is not only built on top of machine learning approaches, but is also designed to work with any RGB camera, making the infrastructure usable on PCs while also guaranteeing high-end performance [59]. Therefore, our system configures itself as a tool for the previously mentioned applications thanks to the use of tabletop cameras instead of professional eye trackers in controlled environments, making our approach accessible in any setting. Furthermore, patient compliance, which is the major concern for online EMDR, is guaranteed by a mixture of the constant presence of the therapist on the call during the therapy and the automatic detection of the patient’s engagement with the prescribed eye movements. Our infrastructure shows strong repeatability, even in sub-optimal lighting conditions, while also aiding the therapists by providing reliable eye tracking measurements [33,59]. While in this specific experiment camera positioning and lighting were controlled to have a degree of consistency between subjects, previous experiments showed that our system has the capability of recognizing pupils and measuring gaze direction even with different camera positioning, uneven lighting, and even partial iris occlusion caused by eyeglasses reflection, making it suited to non-controlled environments. Moreover, the distance detection section further optimizes the system by ensuring optimal distancing of the patients from the camera, guaranteeing even better eye tracking results. Finally, our algorithm is robust to both data latency and data leakage, with the use of encryption in data transmission. In order to improve the current system, the possibility of including more accurate image pre-processing to make the pupils’ movements more easily trackable in case of glasses-wearing patients or sub-optimal lighting is suggested. While the current infrastructure is able to discern pupils even in these conditions, a manual check of the environment at the hands of the experimenters is still encouraged, as borderline cases might interfere with the results of the algorithms (e.g., by measuring a higher number of distractions than those actually occurring). In particular, scarce illumination and eye occlusion might interfere with the algorithm’s capability to identify eyes, which could consequently hinder the whole eye-tracking process. Errors in pupil detection can lead to iris center oscillation, which, while allowed by our system within certain limits, could be wrongly interpreted as non-adherence of the patient to the BLS in cases of excessive oscillations. This is also reflected in the distance detection segment, where the inability to detect the iris could potentially lead to challenges in performing position adjustments at the beginning of the session. Therefore, the main future improvement lies in the machine learning techniques employed for eye tracking, either switching to a full neural network approach to further refine the obtained results or performing more accurate image processing to tackle illumination problems depending on frame luminosity. While these technical considerations are significant, broader limitations of the overall approach must also be acknowledged to provide a balanced perspective. The reliance on eye-tracking technology, although robust under controlled conditions, may face challenges in more variable real-world settings, such as those with inconsistent lighting, hardware differences, or the use of non-ideal equipment. These are hurdles that can be addressed with continued refinement and adaptation of the system but highlight areas where improvements are needed. Another important limitation is the assumption that remote therapy, even when augmented by AI, can fully replicate the depth of interaction found in in-person therapy. While the system ensures the therapist’s active involvement and offers tools to enhance engagement, certain subtle dynamics of physical co-presence, which may play a role in some therapeutic contexts, are inherently difficult to reproduce in virtual settings. However, the accessibility and flexibility provided by the remote approach are transformative, enabling treatment for patients who might otherwise remain untreated, making this trade-off justifiable. Moreover, the scalability of the system across diverse populations and cultural contexts requires further validation. The algorithms have been optimized for specific conditions, and ensuring their effectiveness in broader, heterogeneous groups will be a critical step in advancing this technology. Finally, the approach relies on the availability of stable internet access and compatible technological infrastructure, which could inadvertently exclude patients in under-resourced or remote areas. Addressing this limitation will require parallel efforts to bridge the digital divide and ensure inclusivity. Despite these challenges, the significance of this work remains undiminished. These limitations highlight opportunities for future development, such as advancing machine learning techniques for eye tracking, improving image processing methods to adapt to diverse environmental conditions, and expanding validation efforts to broader populations. By addressing these aspects, the system’s reliability and generalizability can be further enhanced, solidifying its role as a transformative tool for making EMDR therapy accessible to a wider range of patients and contexts.

## 5. Conclusions

The results of this study demonstrate that there is no significant difference in the efficacy of EMDR therapy when delivered in-person versus online using an AI-powered platform under a similar distribution of population; therefore, confirming our hypothesis that the actuation of EMDR in an online setting does not provide worse results than a standard in-presence session. Both groups showed significant reductions in SUDs, PCL-5, and IES-R scores from pre-treatment to post-treatment, with comparable levels of improvement even in terms of statistical distribution on a wide sample of population. These findings are very promising as they suggest that a fully virtual setting, with the active presence and support of the therapist, is able to induce benefits in patients comparable to those obtained with classical therapy, consolidating our approach as a valid and applicable alternative to in-person administration. The successful implementation of the EMDR protocol in an online format with an AI-supported system, such as the one presented in this work, opens up new possibilities for delivering effective mental health interventions to a broader population, particularly in situations where in-person therapy is not feasible.

## Figures and Tables

**Table 1:** Comparison between values pre-treatment, post-treatment and difference pre–post for our three used metrics (SUDs, PCL-5, IES-R). We performed statistical significance analysis with ANOVA (F and *p*) for all nine combinations of metric and IP/OL scores.

		Pre		Post		Δ		Effect Size (Cohen’s d)	ANOVA	
		m	SD	m	SD	m	SD		F	*p*
SUDs (in-person)		8.37	1.43	1.82	1.46	6.55	2.13	1.85	1557.13	<$10^{-4}$
SUDs (online)		8.55	1.30	1.75	1.43	6.80	2.09	2.17	1568.14	<$10^{-4}$
Difference (Cohen’s d)		0.05		0.02		0.04				
ANOVA	F	0.44		0.13		1.51				
	*p*	0.50		0.72		0.22				
PCL-5 (in-person)		28.43	15.55	8.38	7.49	20.20	13.01	1.16	263.05	<$10^{-4}$
PCL-5 (online)		30.37	15.89	8.62	6.89	21.96	13.78	1.25	271.08	<$10^{-4}$
Difference (Cohen’s d)		<0.01		<0.01		<0.01				
ANOVA	F	0.59		0.08		0.90				
	*p*	0.44		0.78		0.35				
IES-R (in-person)		38.41	17.24	11.01	9.39	27.90	16.65	1.39	368.71	<$10^{-4}$
IES-R (online)		40.08	16.28	11.68	8.52	28.76	14.85	1.54	413.59	<$10^{-4}$
Difference (Cohen’s d)		0.07		0.05		0.04				
ANOVA	F	0.51		0.19		0.09				
	*p*	0.48		0.66		0.76				

## Data Availability

The data presented in this study are available on request from the corresponding author. The data are not publicly available in order to comply with the data privacy and protection regulation (mandatory for this experiment).
